# Design Novel Dual Agonists for Treating Type-2 Diabetes by Targeting Peroxisome Proliferator-Activated Receptors with Core Hopping Approach

**DOI:** 10.1371/journal.pone.0038546

**Published:** 2012-06-07

**Authors:** Ying Ma, Shu-Qing Wang, Wei-Ren Xu, Run-Ling Wang, Kuo-Chen Chou

**Affiliations:** 1 Tianjin Key Laboratory on Technologies Enabling Development of Clinical Therapeutics and Diagnostics (Theranostics), School of Pharmacy, Tianjin Medical University, Tianjin, China; 2 Tianjin Institute of Pharmaceutical Research (TIPR), Tianjin, China; 3 Gordon Life Science Institute, San Diego, California, United States of America; Semmelweis University, Hungary

## Abstract

Owing to their unique functions in regulating glucose, lipid and cholesterol metabolism, PPARs (peroxisome proliferator-activated receptors) have drawn special attention for developing drugs to treat type-2 diabetes. By combining the lipid benefit of PPAR-alpha agonists (such as fibrates) with the glycemic advantages of the PPAR-gamma agonists (such as thiazolidinediones), the dual PPAR agonists approach can both improve the metabolic effects and minimize the side effects caused by either agent alone, and hence has become a promising strategy for designing effective drugs against type-2 diabetes. In this study, by means of the powerful “core hopping” and “glide docking” techniques, a novel class of PPAR dual agonists was discovered based on the compound GW409544, a well-known dual agonist for both PPAR-alpha and PPAR-gamma modified from the farglitazar structure. It was observed by molecular dynamics simulations that these novel agonists not only possessed the same function as GW409544 did in activating PPAR-alpha and PPAR-gamma, but also had more favorable conformation for binding to the two receptors. It was further validated by the outcomes of their ADME (absorption, distribution, metabolism, and excretion) predictions that the new agonists hold high potential to become drug candidates. Or at the very least, the findings reported here may stimulate new strategy or provide useful insights for discovering more effective dual agonists for treating type-2 diabetes. Since the “core hopping” technique allows for rapidly screening novel cores to help overcome unwanted properties by generating new lead compounds with improved core properties, it has not escaped our notice that the current strategy along with the corresponding computational procedures can also be utilized to find novel and more effective drugs for treating other illnesses.

## Introduction

Diabetes mellitus is a group of metabolic diseases that has been classified as a disease of glucose overproduction by tissues without enough insulin production, or a disease resulting from cells not responding to the insulin in human body [1]. Type-2 diabetes is the most common type among all the diabetes mellitus forms. The risk of developing type-2 diabetes (T2D) increases with age, obesity, cardiovascular disease (hypertension, dyslipidaemia), lack of physical activity, and family history of diabetes. Increasing dramatically in the US and worldwide, type-2 diabetes has reached epidemic scale. There are nearly 50 million individuals (US) and 314 million individuals (worldwide) with the metabolic syndrome [2]. People suffering from overweight or obesity are of huge risk for developing T2D.

Peroxisome Proliferator-Activated Receptor (PPAR) has drawn increased attention as a drug discovery target by regulating glucose and lipid metabolism [3]. PPAR, and its subtypes PPARα and PPARγ, belong to the superfamily of nuclear receptors that function as transcription factors activated by several ligands. PPARs played a vitally important role in treating obesity, atherogenic dyslipidemia, hypertension, and insulin resistance as main therapeutic targets [4]. The primary function of PPARα is to act as regulator responding to transport and degradation of free fatty acids as well as reverse cholesterol transport by peroxisomal and beta-oxidation pathways [5]. A class of lipid-lowering drugs, such as fenofibrate and gemfibrozil, specially activate PPARα [6], [7], [8]. PPARγ played a significant role in transcriptionally regulating lots of physiological pathways, including adipocyte differentiation and glucose homeostasis [9]. Thiazolidinediones (TZDs) are a class of the antidiabetic drugs, which act by activating the special PPARγ [10]. If used alone, although each of the antidiabetic drugs could enhance the insulin sensitivity and hence lower glucose or fatty acid levels in type-2 diabetic patients [9], some side effects would be caused, such as weight gain, fluid accumulation, and pulmonary edema [11].

Recently, new dual agonists have received considerable attention for developing powerful drugs against diabetes. The strategy of dual agonists was aimed to treat both insulin resistance and dyslipidemia [12]. A critical challenge for developing dual agonists is how to identify the receptor subtype selectivity ratio [13].

Many studies have indicated that computational approaches, such as structural bioinformatics [14], [15], molecular docking [16], [17], pharmacophore modelling [18], QSAR techniques [19], [20], [21], [22], [23], [24], as well as a series of user-friendly web-server predictors developed recently, such as GPCR-2L [25] for identifying G protein-coupled receptors and their types, EnzClassPred [26] for predicting enzyme class, iLoc-Euk [27] and iLoc-Hum [28] for predicting subcellular localization of eukaryotic and human proteins, NR-2L [29] and iNR-PhysChem [30] for identifying nuclear receptors and their subfamilies, and HIVcleave [31] for predicting HIV protease cleavage sites in proteins [32], [33], can timely provide very useful information and insights for drug development. The software of “Core Hopping” [34] is another very powerful and cutting-edge computational technique that is particularly useful for de novel drug design [35].

Encouraged by the aforementioned researches in successfully utilizing various computational approaches for drug development, the present study was initiated in an attempt to screen the fragment database for finding new PPAR dual agonists for treating type-2 diabetes. To realize this, the techniques of “core hopping” with glide docking [34], [36] as well as molecular dynamic simulation were utilized to analyze the binding interactions between the agonist and PPARs in hoping that the findings thus obtained may provide useful insights for developing new and powerful agonists against diabetes mellitus.

## Materials and Methods

The L-tyrosine analogue GW409544 was obtained by modifying the structure of farglitazar, a dual agonist for both PPARα and PPARγ [37]. The main difference between GW409544 and farglitazar is that the former contains a vinylogous amide as the L-tyrosine N-substituent [37]. That is why we chose GW409544 as a starting template structure for designing the new PPAR dual agonists.

The representative complex crystal structures of PPARα (PDB ID 1k7l) and PPARγ (PDB ID 1k74) with the same ligand GW409544 [37] were download from the PDB Bank [38], and were to be used for the molecular modelling studies.

All the calculations were carried out on Dell Precision™ T5500 computer with Schrodinger software package [34], [36] and Desmond 2.4 [39].

**Figure 1:**
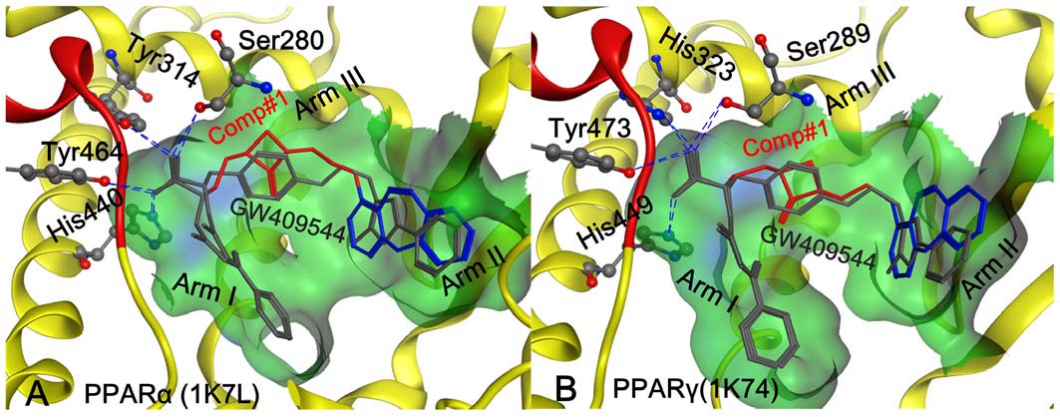
Illustration to show the conformation obtained by docking GW409544 and Comp#1, respectively, to (A) PPARα (1k7l) and (B) PPARγ (1k74). The binding pocket is defined by those residues that have at least one heavy atom with a distance of 5 Å from the ligand [45]. The ligand GW409544 (in grey color) was extracted from the crystal structure while the ligand Comp#1 (rendered by three colors: grey for Core A; red for Core B; and blue for Core C) was generated by the “core-hopping” method. The hydrophobic surface of the receptor is colored in green. The blue dotted lines indicate the H-bond interactions of the receptor with its ligand. The red helix is a part of AF-2 function domain. See the text for further explanation.

### 1. Preparation of Receptor Structures and Databases

The proteins with PDB codes 1k71 and 1k74 were chosen for modeling. In addition to the available knowledge of their 3D (dimensional) structures, the reasons of selecting the two proteins as receptors are as follows. (1) The two proteins contain the same ligand GW409544 as PPARα and PPARγ do, and their binding affinities with the ligand are also quite similar; however, the former selectivity is about 10-fold weaker than the latter [37]. (2) The source organism of both PPARα and PPARγ was from human.

In the process of preparing receptors for modelling, the protein preparation facility [40] was used that included the operations of assigning bond orders, adding hydrogen, treating metals, treating disulfides, deleting waters and alleviating potential steric clashes, adjusting bond order, building missing heavy atom and formal charges, as well as minimizing energy with the OPLS2005 force field [41] and refining the protein by imposing the 0.3 Å RMSD limit as the constraint.

The protein binding-site was identified by the SiteMap tool embedded in Schrodinger Suite 2009 (www.schrodinger.com) as described in [42], [43], [44]. The binding-site encompassed the ligand GW409544, which was observed in the crystal structures of both PPARα (1k71) and PPARγ (1k74) as a ligand.

**Figure 2:**
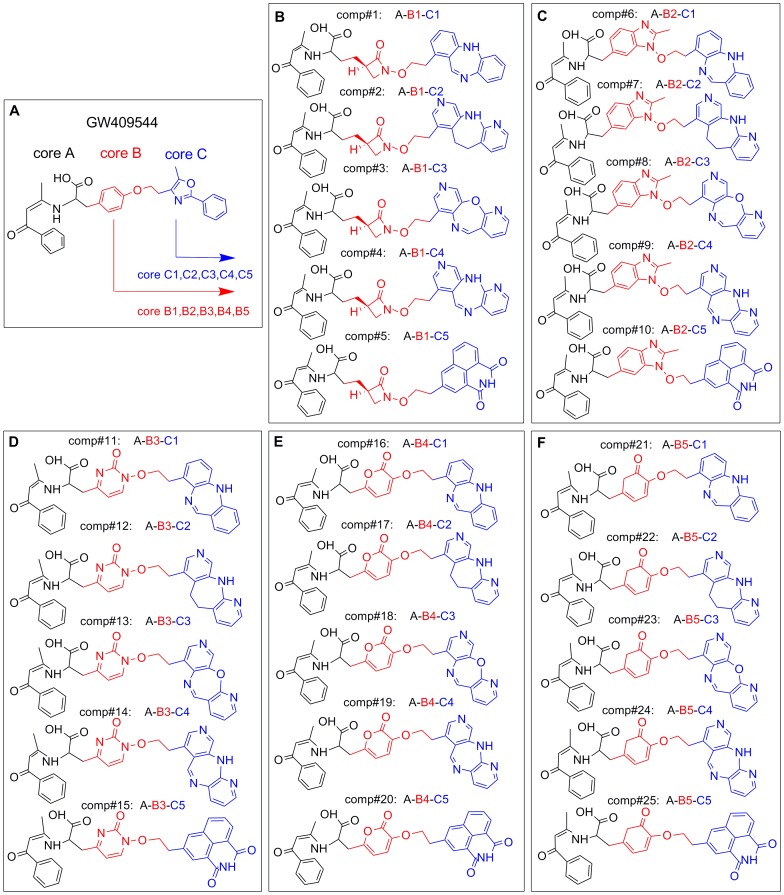
Illustration to show how to generate the 25 derivative compounds from GW409544. (**A**) Structure of GW409544 and its three Cores: Core A (black), Core B (red), and Core C (blue). (**B**) Five compounds derived from GW409544 by changing Core C to C1, C2, C3, C4 and C5 respectively but fixing Core A and Core B at Core B1. Panels (**C**), (**D**), (**E**), and (**F**) each show another five compounds generated by following the similar procedure but fixing Core B at B2, B3, B4, and B5, respectively. See the text for further explanation.

The information of the binding pocket of a receptor for its ligand is very important for drug design, particularly for conducting mutagenesis studies [14]. In the literatures, the binding pocket of a protein receptor to a ligand is usually defined by those residues that have at least one heavy atom (i.e., an atom other than hydrogen) with a distance ≤5 Å from a heavy atom of the ligand. Such a criterion was originally used to define the binding pocket of ATP in the Cdk5-Nck5a* complex [45] that has later proved quite useful in identifying functional domains and stimulating the relevant truncation experiments [46]. The similar approach has also been used to define the binding pockets of many other receptor-ligand interactions important for drug design [15], [17], [47], [48]. In this study, we also used the same criterion [45] to define the binding pockets of proteins 1k7l and 1k74 for the ligand GW409544. A close-up view for the protein-ligand interaction at the binding pocket thus defined is shown in **Fig. 1**, where panel A is for the interaction between PPARα (1k71) and GW409544, while panel B for the interaction between PPARγ (1k74) and GW409544.

Because the natural ligands of PPARs are fatty acids, the binding site of PPARs is almost hydrophobic. Several hydrophobic interactions with three arms of the Y-shaped ligand binding to the site are taken as the key point for designing the new PPARs agonist [49]. The PPAR binding site is composed of three arms, namely Arm I, Arm II, and Arm III, as explicitly marked in **Fig. 1**. The first arm has mainly polar character including the AF2 (transcriptional activation function 2) helix indicated by red ribbon. The hydrophilic head group of the PPAR ligands forms a network of hydrogen bonds with AF2 of Arm-I; while the hydrophobic tail of PPAR agonist is either interacts with Arm II or Arm III. The network hydrogen bonds forms an important conformation for AF2-helix to generate a charge clamp, thus reducing the mobility of AF2 via binding a ligand and hence make it able to regulate the gene expression [4]. The drug-like database and the fragment database derived from ZINC [50] were used for virtual screening and core hopping searching, respectively.

### 2. Molecular Docking with Core Hopping Method

Many useful insights for drug design could be gained via molecular docking studies (see, e.g., [14], [17], [51], [52]). To acquire even more useful information for drug design, a new docking algorithm called “Core Hopping” [36] was adopted in this study that is featured by having the functions to perform both the fragment-based replacing and molecular docking.

Core Hopping [36] is a very powerful and cutting-edge technique for de novel drug design because it can significantly improve the binding affinity of the receptor with its ligands, e.g., GW409544 (**Fig. 2**) in the current study. The binding conformation thus obtained will be taken as an initial structure for further optimization by searching the fragment database to find the optimal cores that are attached to other parts of the template.

During the process of core hopping, the 1st step is to define the possible points at which the cores are attached. It is performed in the “Define Combinations” step from the Combinatorial Screening panel in Schrodinger2009 (www.schrodinger.com). The 2nd step is to define the “receptor grid file”, which was done in the “Receptor Preparation” panel. The 3rd step is the cores preparation that was operated with the “Protocore Preparation” module to find the cores attaching to the scaffold using the fragment database derived from ZINC [50]. The 4th step is to align and dock the entire molecular structure built up by the core and scaffold. The cores are sorted and filtered by goodness of alignment and then redocked into the receptor after attaching the scaffold, followed by using the docking scores to sort the final molecules.

As the products of the core hopping operation, a total of 500 chemical compounds were prepared with the LigPre module [53], which consists of the procedures of generating possible states by ionization at target pH 7.0±2.0, desalting, retaining chiralities from 3D structure and geometry minimization with the OPLS2005 force field [41]. When the above steps were accomplished, all investigated compounds were docked into the receptor pocket through the rigid protein docking model with the Stand-Precision (SP) scoring function [54], [55] to estimate the binding affinities.

**Table 1:** The 25 compounds ranked roughly according to the strength of their docking scores.

Rank	Compound	Combination of cores^a^	Docking scores (Kcal/mol)		ADME properties predicted				
			PPARα (1k71)	PPARγ (1k74)		PSA^b^	logPo/w^c^	logS^d^	PMDCK^e^
	GW409544	A-B-C	−14.41	−15.16		102.39	5.78	−6.80	28.08
1	**Comp#1**	A-B1-C1	−16.10	−16.19		159.92	5.85	−6.53	109.76
2	**Comp#24**	A-B5-C4	−15.87	−16.18		168.65	4.84	−6.50	98.65
3	**Comp#3**	A-B1-C3	−14.89	−16.08		160.40	4.75	−6.59	108.32
4	**Comp#14**	A-B3-C4	−15.10	−15.98		190.08	3.55	−6.28	101.99
5	**Comp#2**	A-B1-C2	−14.97	−15.92		195.19	4.10	−6.81	101.82
6	**Comp#13**	A-B3-C3	−14.68	−15.86		164.26	4.34	−6.16	110.42
7	**Comp#16**	A-B4-C1	−14.26	−15.71		166.47	5.03	−6.92	106.95
8	**Comp#17**	A-B4-C2	−14.44	−15.32		193.75	4.43	−6.43	103.80
9	**Comp#15**	A-B3-C5	−14.42	−15.29		190.02	3.52	−6.57	102.48
10	**Comp#12**	A-B3-C2	−15.15	−15.23		168.27	4.28	−6.81	105.98
11	Comp#19	A-B4-C4	−14.21	−15.25		180.89	4.64	−6.70	109.39
12	Comp#4	A-B1-C4	−14.98	−15.15		185.89	4.23	−7.55	102.25
13	Comp#11	A-B3-C1	−16.15	−15.11		168.04	5.12	−7.74	108.03
14	Comp#25	A-B5-C5	−15.24	−15.09		204.93	3.46	−6.59	101.16
15	Comp#23	A-B5-C3	−15.58	−15.06		176.72	4.89	−7.70	104.35
16	Comp#5	A-B1-C5	−14.77	−15.00		197.47	4.05	−7.30	101.13
17	Comp#21	A-B5-C1	−14.78	−14.98		152.24	6.45	−9.10	106.45
18	Comp#18	A-B4-C3	−13.72	−14.91		186.33	4.43	−7.08	104.19
19	Comp#20	A-B4-C5	−14.42	−14.75		197.70	4.26	−7.20	101.65
20	Comp#9	A-B2-C4	−14.03	−14.35		166.94	5.15	−6.52	114.53
21	Comp#22	A-B5-C2	−15.10	−14.26		150.45	5.55	−8.30	107.48
22	Comp#7	A-B2-C2	−14.16	−13.62		142.49	5.92	−7.32	109.50
23	Comp#6	A-B2-C1	−12.00	−9.14		142.06	6.94	−9.40	120.29
24	Comp#10	A-B2-C5	−11.10	−8.00		174.53	4.59	−7.20	104.59
25	Comp#8	A-B2-C3	−13.07	−7.64		156.10	5.18	−6.25	124.85

a See **Fig. 2** for the structure of cores.

b The van der Waals surface area of polar nitrogen and oxygen atoms (7.0 to 200.0).

c The predicted octanol/water partition coefficient (−2.0 to 6.5).

d The predicted aqueous solubility, where S (mol dm^–3^) is the concentration of the solute in a saturated solution that is in equilibrium with the crystalline solid (−6.5 to 0.5).

e The apparent MDCK permeability (nm/s); MDCK cells are considered to be a good mimic for the bloodbrain barrier. QikProp predictions are for non-active transport (<25 poor; >500 great).

Listed are also their corresponding physiochemical descriptors calculated by QP (QikProp [69]) simulations [68].

### 3. Molecular Dynamics

Many marvelous biological functions in proteins and DNA as well as their profound dynamic mechanisms, such as switch between active and inactive states [56], [57], cooperative effects [58], allosteric transition [59], [60], intercalation of drugs into DNA [61], and assembly of microtubules [62], can be revealed by studying their internal motions [63]. Likewise, to really understand the interaction mechanism of a receptor with its ligand, investigations should be aimed not only at their static structures but also at the dynamic process obtained by simulating their internal motions.

Here, the “Desmond 2.4 Package” [39] was adopted to study the internal motions of the receptor-ligand system. According to the software, the OPLS 2005 force fields [64], [65] was used to build aqueous biological systems, and the TIP3P model [66] was used to simulate water molecules. The orthorhombic periodic boundary conditions were set up to specify the shape and size of the repeating unit. In order to get an electrically neutral system, the minimum number of sodium and chloride ions needed to balance the system charge was placed randomly in the solvated system, and 0.15 mol/L sodium and chloride were then added to mimic the osmotic effect of water. Molecular dynamics simulations were carried out with the periodic boundary conditions in the NPT ensemble. The temperature and pressure were kept at 300 K and 1 atmospheric pressure using Nose-Hoover temperature coupling and isotropic scaling [67]. After all restrains were removed via the 3ns (nanosecond or 10^−9^ of a second) system minimization and relaxation, the operation was followed by running the 10 ns NPT production simulation and saving the configurations thus obtained in 2ps (picosecond or 10^−12^ of a second) intervals. All the molecular dynamics simulations were performed with a time step of 2fs (femtosecond or 10^−15^ of a second).

**Figure 3:**
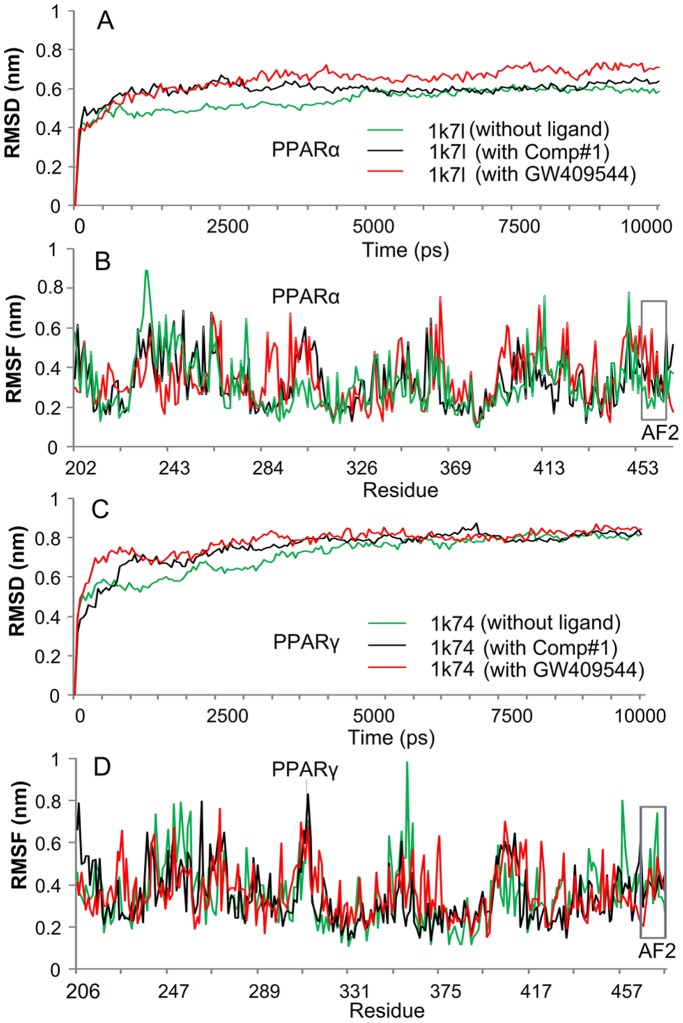
Illustration to show the outcomes of molecular dynamics simulations for Comp#1 ranked number 1 in **Table 1**. (**A**) The RMSD (root mean square deviation) of all backbone atoms for the receptor PPARα. (**B**) The RMSF (root mean square fluctuation) of the side-chain atoms for the receptor PPARα. (**C**) The RMSD (root mean square deviation) of all backbone atoms for the receptor PPARγ. (**D**) The RMSF (root mean square fluctuation) of the side-chain atoms for the receptor PPARγ. The green line indicates the outcome for the system of the receptor alone without any ligand, the red line for that of the receptor with the ligand GW409544, and the black line for that of the receptor with the ligand Comp#1. The curves involved with the AF2 helix region are framed with grey line. See the text for further explanation.

**Figure 4:**
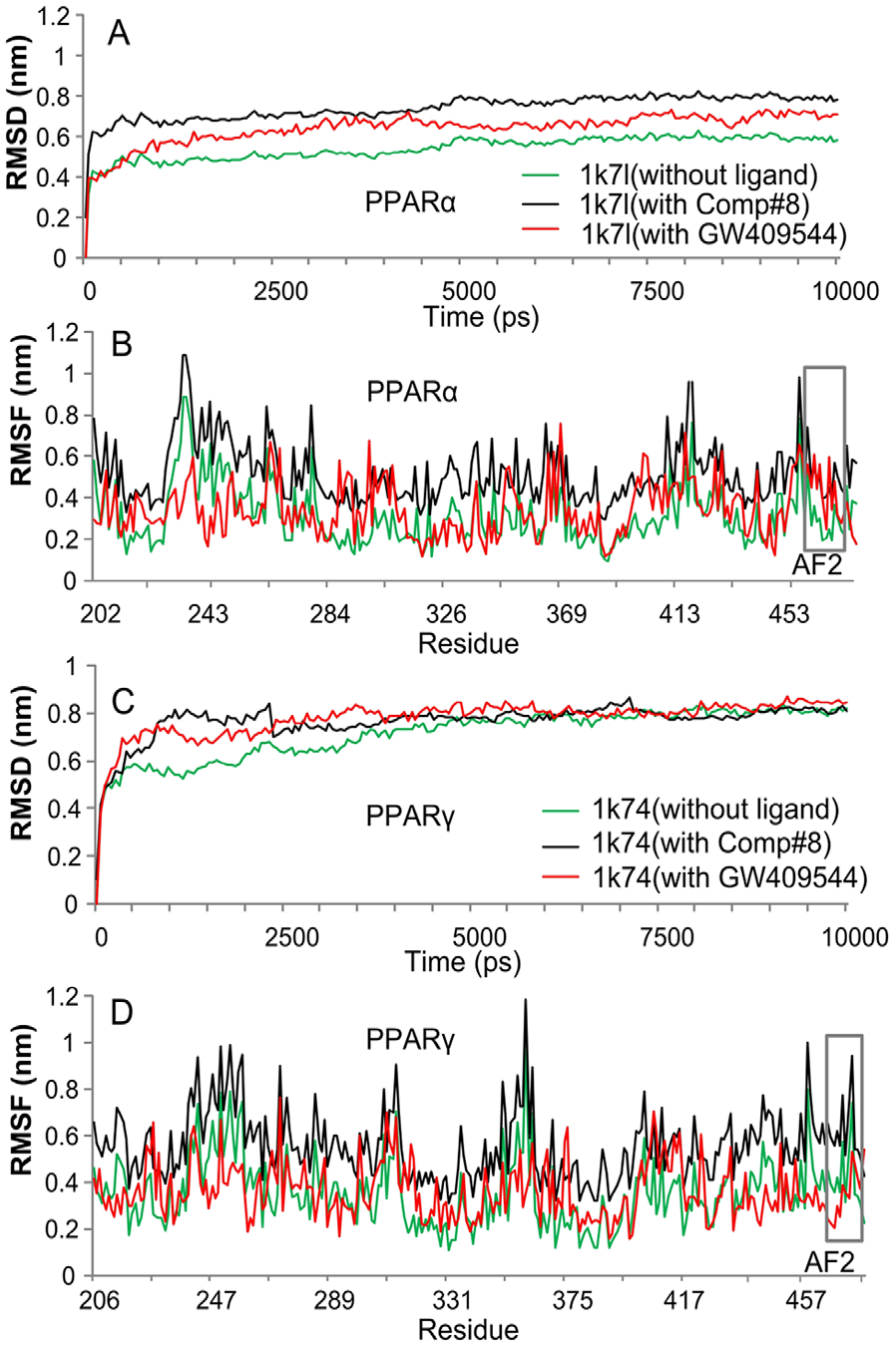
Illustration to show the outcomes of molecular dynamics simulations for Comp#8 ranked number 25 in **Table 1**. (**A**) The RMSD (root mean square deviation) of all backbone atoms for the receptor PPARα. (**B**) The RMSF (root mean square fluctuation) of the side-chain atoms for the receptor PPARα. (**C**) The RMSD (root mean square deviation) of all backbone atoms for the receptor PPARγ. (**D**) The RMSF (root mean square fluctuation) of the side-chain atoms for the receptor PPARγ. The green line indicates the outcome for the system of the receptor alone without any ligand, the red line for that of the receptor with the ligand GW409544, and the black line for that of the receptor with the ligand Comp#8. The curves involved with the AF2 helix region are framed with grey line.

### 4. ADME Prediction

The QikProp [68], [69] is a program for predicting the ADME (absorption, distribution, metabolism, and excretion) properties of the compounds. With the QikProp software, a total of 44 properties of compounds can be predicted, including the principal descriptors and physiochemical properties.

All the compounds investigated need not the treatment for neutralization before using QikProp because it will be automatically done in QikProp. The normal mode was applied in the program. The property analyses for the partition coefficient (QP logP o/w), van der Waals surface area of polar nitrogen and oxygen atoms (PSA), predicted aqueous solubility (QP logS^b^ ), and apparent MDCK permeability (QPP MDCK^c^) [70], were considered in the QikPro to evaluate the acceptability of the compounds.

## Results and Discussion

### 1. Design of PPAR Dual Agonist and Modeling of PPAR Agonist Complex

The process of core hopping and the final agonists’ structures thus selected are illustrated in **Fig. 2**, from which we can see the following. The structure of GW409544, which is conceived as an agonist model to develop novel therapeutic agents for treating metabolic disorder, may be divided into three parts, Core A, Core B, and Core C, as marked by dash lines. Considering the great importance of the acidic head in Core A for activating PPARs receptors, let us retain the Core A part during the core hopping calculation as described below.

The 1st core hopping operation was aimed at the Core C part (see red part of **Fig. 2**), generating five cores, Core C1, Core C2, Core C3, Core C4, and Core C5, respectively, to replace Core C. The 2nd core hopping operation was aimed at the Core B part (see blue part of **Fig. 2**), also respectively generating five cores, Core B1, Core B2, Core B3, Core B4, and Core B5 to replace Core B.

Consequently, we have a total of 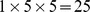 different combinations for the GW409544 derivatives thus generated. Subsequently, each of the 25 derivative compounds was docked into the two receptors PPARα (1k71) and PPARγ (1k74), respectively. Listed in **Table 1** are the 25 derivative compounds ranked roughly according to their docking scores to the receptors PPARα and PPARγ, respectively. The top ten compounds highlighted with boldface type in **Table 1** are those derivatives that are stronger than the original GW409544 in binding affinity with the two receptors. Of the top ten derivatives, the Comp#1, i.e., “Core A-Core B1-Core C1”, has the strongest binding affinity with both PPARα (1k71) and PPARγ (1k74), and hence it was singled out for further investigation.

Shown in **Fig. 1** is the docked conformation of Comp#1 when aligned with GW409544 extracted from (**A**) the crystal complex in PPARα (1k7l) and (**B**) the crystal complex in PPARγ (1k74), respectively.

As described in [37], [71], the conversed H-bonding network formed by the polar acidic head of Core A in both GW409544 and Comp#1 to the four key residues of PPARα (or PPARγ), such as Ser280 (or Ser289), Tyr314 (or His323), Tyr464 (or Tyr473) and His440 (or His449), were observed in our docking study. This H-bonding network played the role in stabilizing the conformation of the AF2-helix in arm I (red helix in **Fig. 1**), which is vitally important for receptor-binding and activation [37], [72], [73], [74], [75], [76], [77].

The hydrophobic tail of both Core A and Core C of agonists are buried well in the hydrophobic arm I and arm II that are formed by hydrophobic residues as shown by the green surface in **Fig. 1**. Compared with GW409544 (shown with grey color in **Fig. 1**), the compound of Comp#1 (purple color in **Fig. 1**) has more bulky molecular volume owing to the large hydrophobic Core C1, which is more fitted to the hydrophobic arm II, resulting in the much better binding affinity than GW409544 (cf. **Table 1**).

### 2. Molecular Dynamics Trajectory Analysis

Molecular dynamics can provide useful information for characterizing the internal motions of biomacromolecules with time. For a comparison study, the 10 ns molecular dynamics simulations were performed, respectively, for the crystal structures of PPARα (1k7l), PPARγ (1k74), as well as their complexes with GW409544 and Comp#1, i.e., PPARα-GW409544, PPARγ-GW409544, PPARα-Comp#1, and PPARγ-Comp#1. As we can see from **Fig. 3**, all the characters concerned reached the simulation equilibrium within the 5ns (see panels **A** and **C**).

Meanwhile, the corresponding root mean square deviation (RMSD) value curves of the protein backbone for PPARα, PPARγ, PPARα-GW409544, PPARγ-GW409544, PPARα-Comp#1, and PPARγ-Comp#1 were also computed, respectively. It is interesting to see that the RMSD curves for PPARα-Comp#1 and PPARγ-Comp#1 are remarkably more stable than those of PPARα-GW409544 and PPARγ-GW409544, particularly for the case of PPARα (1k7l) system, where only a fluctuation of around 0.3 nm was observed when the complex system reached the plateau.

The detailed fluctuations of the aforementioned six different structures, as well as the root mean square fluctuations (RMSF) of their side-chain atoms, were also computed within 10 ns molecular dynamics simulations (see panels **B** and **D** of **Fig. 3**).

It is instructive to point out that the RMSF curve of PPARα-Comp#1 or PPARγ-Comp#1 is highly similar to that of PPARα-GW409544 or PPARγ-GW409544, respectively. This is especially remarkable in the binding site of AF2 helix region with the residues 459–465 for PPARα-Comp#1 and residues 469–477 for PPARγ-Comp#1 (see the grey frames in **Fig. 3B and D**), indicating that the new designed compound, Comp#1, is very likely to have the same function for activating the AF2 helix as done by GW409544.

As a negative control, the similar molecular dynamics simulation was also performed for Comp#8 (A-B2-C3), which is ranked number 25 according to the strength of binding affinity with PPARα and PPARγ (cf. **Table 1**). The corresponding simulation results thus obtained are shown in the **Fig. 4**, from which we can see that the fluctuating magnitudes of molecular dynamics for PPARα-Comp#8 and PPARγ-Comp#8, including the RMSD and RMSF, are much larger than those of PPARα-Comp#1 and PPARγ-Comp#1, especially for the binding site of AF2 helix region (see the gray frames in **Fig. 4B and D**). These phenomena indicate that Comp#8 is not as good as Comp#1 in stably binding to PPARα and PPARγ, and hence Comp#8 might not have the same function for activating the AF2 helix as GW409544 had.

### 3. ADME Prediction

Some pharmaceutically relevant properties of the new designed agonist derivatives as well as the original GW409544 compound, such as the “partition coefficient” (logPo/w), “van der Waals surface area of polar nitrogen and oxygen atoms” (PSA), “aqueous solubility” (logS), and “apparent MDCK permeability” (PMDCK), were predicted by means of the QP program embedded in the “Schrodinger2009 Software Package”. The results thus obtained are also listed in the **Table 1**, respectively. Since PPARα and PPARγ have a more spacious pocket (∼1400 Å^3^) than any other nuclear hormone receptors [37], [72], it is quite natural that the agonist derivatives designed based on the two receptors by combining their three cores would have relatively large molecular weight (MW>500) and bulky volume, a trend quite similar to case in designing the inhibitors against the protein tyrosine phosphates (PTPase) [78].

As shown in **Table 1**, the values calculated by the QP program, such as PAS, logPo/w, logS, and PMDCK for the newly designed agonists are all within the reasonable ranges. Although the higher logPo/w value of a compound, the stronger its affinity to PPARs is, it is not a good idea to excessively enhance logPo/w because this would induce bad distribution of the compound on fat and body fluid [70]. It should be pointed out that, rather than the experiential values within the range between −6.5 and 0.50, most of the log S values for the new agonists are quite close to that of GW409544. Such a phenomenon might result from the core A part which was kept unchanged during the process of designing the newly compounds as mentioned above. If the core A part was modified as well, the log S value would be further improved accordingly. Also, as mentioned above, the values for the four ADME properties listed in **Table 1** are all within the acceptable range for human beings, indicating that most of the 25 compounds, particularly the top 10 derivatives found in this study as highlighted in **Table 1**, can be utilized as candidates for the purpose of developing new drugs.

### 4. Conclusions

The goal of this study was to find new and more powerful dual agonists for PPARα and PPARγ. The new technique of “core hopping” adopted in this study allows for the rapid screening of novel cores to help overcome unwanted properties by generating new lead compounds with improved core properties. A set of 10 novel compounds were found in this regard. Compared with the existing dual agonist, the new agonists not only had the similar function in activating PPARα and PPARγ, but also assumed the conformation more favorable in binding to PPARα and PPARγ. It is anticipated that the new agonists may become potential drug candidates. Or at the very least, they may stimulate new strategy for developing novel dual agonists against type-2 diabetes.
